# Supplementary material for: Contextual AI models for single-cell protein biology
Source: Nat Methods. 2024 Jul 22;21(8):1546–57. doi: 10.1038/s41592-024-02341-3 (PMC11310085; doi:10.1038/s41592-024-02341-3)
Supplement: Supplementary file 1 — Supplementary Notes 1–3, Tables 1–6 and references. [file 41592_2024_2341_MOESM1_ESM.pdf]

nature methods

Supplementary information https://doi.org/10.1038/s41592-024-02341-3

Contextual AI models for single-cell protein biology

In the format provided by the authors and unedited

Contents

S1 Supplementary Notes **S2**

S1.1 Supplementary Note S1: Analyzing PINNACLE's embeddings for proteins with similar function S2

S1.2 Supplementary Note S2: Benchmarking contextual 3D structure-based protein representations S3

S1.3 Supplementary Note S3: Extending PINNACLE to generate cell-level embeddings S4

S2 Supplementary Tables **S5**

S3 Supplementary References **S9**

S1 Supplementary Notes

S1.1 Supplementary Note S1: Analyzing PINNACLE's embeddings for proteins with similar function

Dataset. We extract human housekeeping genes from the Housekeeping and Reference Transcript Atlas (https://housekeeping.unicamp.br/)[1] and marker genes from the human gold standard T lymphocyte-specific protein functional networks from HumanBase (https://hb.flatironinstitute.org/) (accessed on November 20th, 2023)[2]. From HumanBase, only edges of level C1 (i.e., tissue-specific) are kept. The nodes corresponding to these edges are considered to be marker genes for cell types in the family of T lymphocytes. The lists of marker and housekeeping genes do not overlap, as we remove any overlapping housekeeping genes from the list of marker genes.

Analysis. We compare embedding similarities of a marker (orange) or housekeeping (gray) gene's contextualized protein representation (from PINNACLE) across different cell type contexts. For each marker or housekeeping gene, its cell type-specific protein representations are compared in similar contexts (i.e., between different T lymphocyte cell types; a total of 10 T lymphocyte cell types) or different contexts (i.e., between a T lymphocyte cell type and a non-immune cell type; a total of 115 non-immune cell types). We perform the two-sample Kolmogorov-Smirnov test (via `ks_2samp` from `scipy`).

Results. Although PINNACLE learns protein representations using context-aware protein, cell type, and tissue networks alone, it effectively captures protein functions. We analyze the embedding similarities of contextualized protein representations for marker and housekeeping genes across cell type contexts. For each T lymphocyte marker or housekeeping gene, we compare its cell type-specific protein representations in similar contexts (i.e., between different T lymphocyte cell types) and in different contexts (i.e., between a T lymphocyte cell type and a non-immune cell type). Housekeeping genes exhibit higher embedding similarity in similar contexts than marker genes (Supplementary Figure S5; p-value $= 3.2 \times 10^{-14}$). This result aligns with the expectation that housekeeping genes maintain shared functions across these cell types. Housekeeping genes in different contexts also show higher embedding similarity than marker genes (Supplementary Figure S5; p-value $= 1.0 \times 10^{-91}$), reflecting their consistent functions across non-immune cell types. Conversely, marker genes in similar contexts display higher embedding similarity than those in different contexts (Supplementary Figure S5; p-value $= 3.1 \times 10^{-26}$), consistent with their specificity to T lymphocyte cell types. Their protein representations are more similar within T lymphocyte contexts compared to when these marker genes are in the context of non-immune cell types. These analyses suggest that the protein embedding regions in PINNACLE are organized according to cellular contexts, potentially capturing subtle nuances not explicitly included in the training dataset or the model itself. This encompasses the possibility of cell type-dependent roles for proteins, a complexity that can enhance our understanding of protein functions across different biological contexts. Such insights warrant further investigation into proteins with context-specific and non-specific functions.

S1.2 Supplementary Note S2: Benchmarking contextual 3D structure-based protein representations

Results. We benchmark our contextualized protein representations (structure-free) and contextualized structure-based protein representations against two null distributions and four context-free approaches. We show that randomly sampling pairs of proteins from different cell type contexts, padded (no 3D structure; score gap -0.0431) or concatenated with the structure-based protein representations (score gap -0.0356), cannot produce the score gap observed in the contextualized protein representations (PINNACLE without 3D structure) nor contextualized structure-based protein representations (PINNACLE with 3D structure) (Supplementary Figure S7). Similarly, context-free protein representations cannot predict intercellular communication (i.e., protein interactions between different cell types). Such is demonstrated using context-free protein representations generated by a graph attention neural network[3] on the global reference protein interaction network (i.e., GAT), padded (no 3D structure; score gap -0.1319) and concatenated with the structure-based protein representations (score gap -0.0486), and context-free protein representations generated by BIONIC[4], a graph convolutional neural network designed for multi-modal network integration, padded (score gap 0.0046) and concatenated with the structure-based protein representations (score gap 0.0043). Our benchmarking results suggest that incorporating context can improve 3D structure prediction of protein interactions.

S1.3 Supplementary Note S3: Extending PINNACLE to generate cell-level embeddings

Unlike approaches that generate cell embeddings to advance cell-level downstream tasks, such as batch correction and cell type annotation[5–7], PINNACLE generates protein representations across cell types for precise protein-level prediction at cell type resolution. PINNACLE learns embeddings of cell types and tissues as a means to inject cellular and tissue organization (via the metagraph) into the unified protein embedding space. To enable cell-level characterization, PINNACLE can easily be extended to learn cell (rather than cell *type*) embeddings.

S2 Supplementary Tables

Table S1: Predicted ranks of relevant cell types for rheumatoid arthritis. Shown are predicted ranks of subtypes of T cells, natural killer (NK) cells, dendritic cells, B cells, monocytes, and myeloid cells. These cell types have been demonstrated in existing literature to be involved with rheumatoid arthritis[8–10].

**Cell type**	**Subtypes**	**PINNACLE-Predicted Rank**
T cell	CD4+ helper T cell	1
	CD4+ $\alpha\beta$ memory T cell	2
	Regulatory T cell	6
	CD8+ $\alpha\beta$ cytotoxic T cell	27
	DN1 thymic pro-T cell	33
	Mature natural killer T cell	34
	Naive regulatory T cell	39
	Type I natural killer T cell	49
	Naive thymus-derived CD4+ $\alpha\beta$ T cell	77
	CD8+ $\alpha\beta$ cytokine secreting effector T cell	104
Dendritic cell	CD1c+ myeloid dendritic cell	3
	CD141+ myeloid dendritic cell	11
	Dendritic cell	37
	Mature conventional dendritic cell	38
	Myeloid dendritic cell	42
	Plasmacytoid dendritic cell	43
	Liver dendritic cell	50
B cell	Memory B cell	12
	B cell	29
Natural killer cell	Natural killer cell	17
	Immature natural killer cell	113
Monocyte	Intermediate monocytes	18
	Non-classical monocytes	68
	Monocyte	85
	Classical monocytes	91
Myeloid cell	Myeloid progenitor	71
	Myeloid cell	95

Table S2: Predicted ranks of relevant cell types for inflammatory bowel diseases. Shown are predicted ranks of subtypes of T cell, fibroblast, goblet cell, enterocyte, monocyte, natural killer cell, B cell, glial cell, dendritic cell, and macrophage. These cell types have been demonstrated in existing literature to be involved with inflammatory bowel diseases[11,12].

Cell type	Subtypes	PINNACLE-Predicted Rank
T cell	CD4+ $\alpha\beta$ memory T cell	1
	Naive thymus-derived CD4+ $\alpha\beta$ T cell	11
	Regulatory T cell	14
	DN1 thymic pro-T cell	15
	Mature natural killer T cell	16
	CD8+ $\alpha\beta$ cytokine secreting effector T cell	19
	Type I natural killer T cell	30
	CD4+ helper T cell	41
	Naive regulatory T cell	43
	CD8+ $\alpha\beta$ cytotoxic T cell	56
Enterocyte	Enterocyte of epithelium of large intestine	2
	Mature enterocyte	22
	Immature enterocyte	90
	Enterocyte of epithelium of small intestine	94
	Intestinal enteroendocrine cell	138
Dendritic cell	Myeloid dendritic cell	5
	Dendritic cell	10
	CD1c+ myeloid dendritic cell	13
	CD141+ myeloid dendritic cell	50
	Liver dendritic cell	54
	Mature conventional dendritic cell	67
	Plasmacytoid dendritic cell	72
Goblet cell	Large intestine goblet cell	7
	Goblet cell	33
	Small intestine goblet cell	75
	Respiratory goblet cell	109
	Tracheal goblet cell	110
B cell	B cell	9
	Memory B cell	45
Monocyte	Intermediate monocyte	26
	Non-classical monocyte	81
	Monocyte	89
	Classical monocyte	93
Glial cell	Microglial cell	29
	Radial glial cell	107
Natural killer cell	Immature natural killer cell	31
	Natural killer cell	73
Fibroblast	Fibroblast	49
Macrophage	Macrophage	74

Table S3: Metagraph network statistics with different cutoff selection. Data statistics of the metagraph with different cutoffs for the minimum number of significant ligand-receptor interactions between a pair of cell types to create an edge. Shown are the number of nodes, number of edges, and the average degree of the cell type-cell type interaction (CCI) graph and the metagraph (includes cell type-cell type, cell type-tissue, and tissue-tissue edges).

**Cutoff for Significant LRs**	**Component of Metagraph**	**Number of Nodes**	**Number of Edges**	**Average Degree**
Cutoff = 1 (Original)	CCI Graph	156	3,567	45.7
	Metagraph (All)	218	4,018	36.9
Cutoff = 2	CCI Graph	156	1,808	23.2
	Metagraph (All)	218	2,259	20.7
Cutoff = 3	CCI Graph	156	1,736	22.3
	Metagraph (All)	218	2,187	20.1
Cutoff = 4	CCI Graph	156	1,640	21.0
	Metagraph (All)	218	2,091	19.2
Cutoff = 5	CCI Graph	156	1,576	20.2
	Metagraph (All)	218	2,027	18.6

Table S4: Sensitivity analysis of cutoff selection. Sensitivity analysis to examine the impact of the cutoff value for the minimum required number of significant ligand-receptor interactions in the cell-type-to-cell-type graph on PINNACLE's embedding space. The first row consists of results from the complete model. The remaining four rows show results from cutoff values 2, 3, 4, and 5. All Spearman correlation statistical tests are two-sided.

**Model**	**Tissue Embedding Distance vs. Ontology Distance**	**Tissue Embedding Distance vs. Fraction of Cell Type Overlap (leaves only)**
Complete model (Cutoff = 1)	Spearman $\rho = 0.36$ p-value $= 4.6 \times 10^{-119}$	Spearman $\rho = -0.46$ p-value $= 8.01 \times 10^{-30}$
Cutoff = 2	Spearman $\rho = 0.21$ p-value $= 1.0 \times 10^{-37}$	Spearman $\rho = -0.31$ p-value $= 2.4 \times 10^{-13}$
Cutoff = 3	Spearman $\rho = 0.22$ p-value $= 1.7 \times 10^{-41}$	Spearman $\rho = -0.31$ p-value $= 7.8 \times 10^{-14}$
Cutoff = 4	Spearman $\rho = 0.25$ p-value $= 1.4 \times 10^{-53}$	Spearman $\rho = -0.29$ p-value $= 2.3 \times 10^{-12}$
Cutoff = 5	Spearman $\rho = 0.38$ p-value $= 8.8 \times 10^{-129}$	Spearman $\rho = -0.25$ p-value $= 3.7 \times 10^{-9}$

Table S5: Ablation studies to interrogate the contribution of the metagraph. The first row consists of results from the complete model. The remaining three rows show results from three types of ablations: removing cell-type-to-cell-type relationships (i.e., shuffling the cell type nodes' identities), removing tissue-to-tissue relationships (i.e., shuffling the tissue nodes' identities), and removing the metagraph (i.e., setting the weight of the metagraph-related terms in the loss function to zero). The performance metrics evaluate the models' ability to capture cell type and tissue organization in the embedding space. The second column is the correlation between tissue embedding distance (computed using the model's tissue representations) and tissue ontology distance; we expect a positive correlation. The third column is the correlation between tissue embedding distance and tissue ontology distance among the tissue leaf nodes of the metagraph; we expect a positive correlation. The fourth column is the correlation between tissue embedding distance and fraction of overlapping cell types; we expect a strong negative correlation. All Spearman correlation statistical tests are two-sided.

**Model**	**Tissue Embedding Distance vs. Ontology Distance**	**Tissue Embedding Distance vs. Ontology Distance (leaves only)**	**Tissue Embedding Distance vs. Fraction of Cell Type Overlap (leaves only)**
Complete model	Spearman $\rho = 0.36$<br>p-value $= 4.6 \times 10^{-119}$	Spearman $\rho = 0.11$<br>p-value $= 0.01$	Spearman $\rho = -0.46$<br>p-value $= 8.01 \times 10^{-30}$
Drop cell type-cell type graph	Spearman $\rho = 0.38$<br>p-value $= 2.3 \times 10^{-132}$	Spearman $\rho = 0.10$<br>p-value $= 0.02$	Spearman $\rho = -0.21$<br>p-value $= 4.25 \times 10^{-7}$
Drop tissue-tissue graph	Spearman $\rho = -0.13$<br>p-value $= 1.2 \times 10^{-14}$	Spearman $\rho = -0.15$<br>p-value $= 6.5 \times 10^{-4}$	Spearman $\rho = -0.16$<br>p-value $= 2.5 \times 10^{-4}$
Drop metagraph loss	Spearman $\rho = 0.30$<br>p-value $= 4.6 \times 10^{-79}$	Spearman $\rho = -0.10$<br>p-value $= 0.02$	Spearman $\rho = -0.19$<br>p-value $= 1.1 \times 10^{-5}$

Table S6: Data split of downstream tasks. Sizes of the train, validation, and test datasets for the rheumatoid arthritis (RA) PINNACLE model and inflammatory bowel disease (IBD) PINNACLE model. The numeric value outside the parentheses represents the number of protein representations across cell type contexts, and the numeric value inside the parentheses represents the number of unique protein identities. The numbers represent both positive (label = 1) and negative (label = 0) proteins. Note that the validation dataset set is sampled from the train dataset, which is fixed, at each run of the model. The numbers for train and validation datasets (columns 3-4) are from seed 1.

**Dataset**	**Type of protein target**	**Proteins in train dataset (unique)**	**Proteins in validation dataset (unique)**	**Proteins in test dataset (unique)**
RA	Total	17,408 (600)	6,647 (195)	25,137 (818)
	Positive	1,319 (53)	570 (21)	2,226 (78)
	Negative	16,089 (547)	6,077 (174)	22,911 (740)
IBD	Total	27,652 (896)	9,363 (297)	8,864 (294)
	Positive	1,210 (62)	673 (26)	731 (26)
	Negative	26,442 (834)	8,690 (271)	8,133 (268)

S3 Supplementary References

1. Hounkpe, B. W., Chenou, F., de Lima, F. & De Paula, E. V. HRT Atlas v1. 0 database: redefining human and mouse housekeeping genes and candidate reference transcripts by mining massive RNA-seq datasets. *Nucleic Acids Research* **49**, D947–D955 (2021).

2. Greene, C. S. *et al.* Understanding multicellular function and disease with human tissue-specific networks. *Nature Genetics* **47**, 569–576 (2015).

3. Brody, S., Alon, U. & Yahav, E. How attentive are graph attention networks? *ICLR* (2022).

4. Forster, D. T. *et al.* BIONIC: biological network integration using convolutions. *Nature Methods* **19**, 1250–1261 (2022).

5. Chen, H., Ryu, J., Vinyard, M. E., Lerer, A. & Pinello, L. SIMBA: single-cell embedding along with features. *Nature Methods* 1–11 (2023).

6. Theodoris, C. V. *et al.* Transfer learning enables predictions in network biology. *Nature* **618**, 616–624 (2023).

7. Cui, H. *et al.* scGPT: toward building a foundation model for single-cell multi-omics using generative AI. *Nature Methods* 1–11 (2024).

8. Lewis, M. J. *et al.* Molecular portraits of early rheumatoid arthritis identify clinical and treatment response phenotypes. *Cell Reports* **28**, 2455–2470 (2019).

9. Zhang, F. *et al.* Deconstruction of rheumatoid arthritis synovium defines inflammatory subtypes. *Nature* **623**, 616–624 (2023).

10. Vickovic, S. *et al.* Three-dimensional spatial transcriptomics uncovers cell type localizations in the human rheumatoid arthritis synovium. *Communications Biology* **5**, 129 (2022).

11. Smillie, C. S. *et al.* Intra-and inter-cellular rewiring of the human colon during ulcerative colitis. *Cell* **178**, 714–730 (2019).

12. Kong, L. *et al.* The landscape of immune dysregulation in Crohn's disease revealed through single-cell transcriptomic profiling in the ileum and colon. *Immunity* **56**, 444–458 (2023).
