# Supplementary material for: Contextual AI models for single-cell protein biology
Source: Nat Methods. 2024 Jul 22;21(8):1546–57. doi: 10.1038/s41592-024-02341-3 (PMC11310085; doi:10.1038/s41592-024-02341-3)
Supplement: Supplementary file 2 — Reporting Summary [file 41592_2024_2341_MOESM2_ESM.pdf]

nature portfolio

Corresponding author(s): Zitnik, Marinka

Last updated by author(s): May 9, 2024

Reporting Summary

Nature Portfolio wishes to improve the reproducibility of the work that we publish. This form provides structure for consistency and transparency in reporting. For further information on Nature Portfolio policies, see our Editorial Policies and the Editorial Policy Checklist.

Statistics

For all statistical analyses, confirm that the following items are present in the figure legend, table legend, main text, or Methods section.

n/a	Confirmed	
☐	☒	The exact sample size (*n*) for each experimental group/condition, given as a discrete number and unit of measurement
☒	☐	A statement on whether measurements were taken from distinct samples or whether the same sample was measured repeatedly
☐	☒	The statistical test(s) used AND whether they are one- or two-sided<br>*Only common tests should be described solely by name; describe more complex techniques in the Methods section.*
☐	☒	A description of all covariates tested
☐	☒	A description of any assumptions or corrections, such as tests of normality and adjustment for multiple comparisons
☐	☒	A full description of the statistical parameters including central tendency (e.g. means) or other basic estimates (e.g. regression coefficient) AND variation (e.g. standard deviation) or associated estimates of uncertainty (e.g. confidence intervals)
☐	☒	For null hypothesis testing, the test statistic (e.g. *F*, *t*, *r*) with confidence intervals, effect sizes, degrees of freedom and *P* value noted<br>*Give P values as exact values whenever suitable.*
☒	☐	For Bayesian analysis, information on the choice of priors and Markov chain Monte Carlo settings
☒	☐	For hierarchical and complex designs, identification of the appropriate level for tests and full reporting of outcomes
☐	☒	Estimates of effect sizes (e.g. Cohen's *d*, Pearson's *r*), indicating how they were calculated

Our web collection on statistics for biologists contains articles on many of the points above.

Software and code

Policy information about availability of computer code

Data collection

Python implementation of the methodology developed and used in the study is available via the project website at https://zitniklab.hms.harvard.edu/projects/PINNACLE. The code to reproduce results, together with documentation and examples of usage, are available on GitHub at https://github.com/mims-harvard/PINNACLE. We provide an interactive demo via HuggingFace to explore PINNACLE's contextualized protein representations

Data analysis

Python implementation of the methodology developed and used in the study is available via the project website at https://zitniklab.hms.harvard.edu/projects/PINNACLE. The code to reproduce results, together with documentation and examples of usage, are available on GitHub at https://github.com/mims-harvard/PINNACLE. We provide an interactive demo via HuggingFace to explore PINNACLE's contextualized protein representations.

We visualize PINNACLE embeddings using a uniform manifold approximation and projection for dimension reduction (UMAP package version 0.5, https://umap-learn.readthedocs.io/en/latest/) and Seaborn (seaborn package version 0.13, https://seaborn.pydata.org/index.html). Additionally, we use the Python implementation of SAFE version 1, https://github.com/baryshnikova-lab/safepy.

We implement PINNACLE using Pytorch (Version 1.12.1) (Paszke et al., 2019) and Pytorch Geometric (Version 2.1.0) (Fey et al., 2019). We leverage Weights and Biases (Biewald 2020) for hyperparameter tuning and model training visualization, and we create interactive demos of the model using Gradio (Abid et al. 2019).

For manuscripts utilizing custom algorithms or software that are central to the research but not yet described in published literature, software must be made available to editors and reviewers. We strongly encourage code deposition in a community repository (e.g. GitHub). See the Nature Portfolio guidelines for submitting code & software for further information.

Data

Policy information about availability of data

All manuscripts must include a data availability statement. This statement should provide the following information, where applicable:

- Accession codes, unique identifiers, or web links for publicly available datasets
- A description of any restrictions on data availability
- For clinical datasets or third party data, please ensure that the statement adheres to our policy

All data used in the paper are shared via the project website at https://zitniklab.hms.harvard.edu/projects/PINNACLE.

Our global reference protein-protein interaction (PPI) network is the union of physical multi-validated interactions from BioGRID (Oughtred et al., 2019), the Human Reference Interactome (HuRI) (Luck et al., 2020), and Menche et al., 2015 with 15,461 nodes and 207,641 edges. Different sources of PPI have their own methods of curating and validating physical interactions between proteins. BioGRID, HuRI, and Menche et al. are PPI networks from three well-cited publications and databases regarding human protein interactions. By joining the three networks, we construct a comprehensive global PPI network for our analysis.

We leverage Tabula Sapiens (Tabula Sapiens Consortium, 2022) data source as our multi-organ, single-cell transcriptomic atlas of humans. The data consists of 15 donors, with 59 specimens total. There are 483,152 cells after quality control, of which 264,824 are immune cells, 104,148 are epithelial cells, 31,691 are endothelial cells, and 82,478 are stromal cells. The cells correspond to 177 unique cell ontology classes.

For 3D structural analyses, the proteins being compared are PD-1, PD-L1, B7-1, CTLA-4, RalB, RalBP1, EPO, EPOR, C3, and CFH. The pairs of binding proteins are PD-1/PD-L1 (PDB ID: 4ZQK) and B7-1/CTLA-4 (PDB ID: 1I8L). The non-binding proteins are any of the four proteins paired with any of the remaining six proteins (e.g., PD-1/RalB, PD-1/RalBP1, PD-L1/RalBP1). The PDB IDs for the other six proteins are 2KWI for RalB/RalBP1, 1CN4 for EPO/EPOR, and 3OXU for C3/CFH.

We obtain labels for therapeutic targets from the Open Targets Platform (Ochoa et al., 2020).

Human research participants

Policy information about studies involving human research participants and Sex and Gender in Research.

Reporting on sex and gender	This study did not involve human research participants.
Population characteristics	This study did not involve human research participants.
Recruitment	This study did not involve human research participants.
Ethics oversight	This study did not involve human research participants.

Note that full information on the approval of the study protocol must also be provided in the manuscript.

Field-specific reporting

Please select the one below that is the best fit for your research. If you are not sure, read the appropriate sections before making your selection.

☒ Life sciences ☐ Behavioural & social sciences ☐ Ecological, evolutionary & environmental sciences

For a reference copy of the document with all sections, see nature.com/documents/nr-reporting-summary-flat.pdf

Life sciences study design

All studies must disclose on these points even when the disclosure is negative.

Sample size

No statistical methods were used to determine sample sizes. Small 95% confidence intervals or standard deviations indicate the chosen sample sizes were sufficient.

1) Cell type-specific protein interaction network samples. To ensure high-quality representations of cell types in our networks, we keep networks with at least 1,000 proteins. We do not perform subsampling of cells (i.e., sample the same number of cells per cell type) to minimize information loss for constructing protein interaction networks (Supplementary Figure S2).

2) Cell type and tissue relationship samples in the metagraph. As recommended by CellPhoneDB, cells are subsampled prior to running the algorithm, which uses geometric sketching [105] to efficiently sample a small representative subset of cells from massive datasets while preserving biological complexity. We choose to subsample 25% of cells and run CellPhoneDB for 100 iterations. We determine cell type-tissue relationships and extract tissue-tissue relationships using Tabula Sapiens meta-data. For relationships between cell types and tissues, we draw edges between cell types and the tissue that the cells were taken from. For tissue-tissue relationships, we select the nodes corresponding to the tissues where samples were taken from and all parent nodes up to the root of the BRENDA tissue ontology. We perform sensitivity and ablation analyses on different components of the metagraph (Supplementary Table S3-S5).

3) Therapeutic area selection. To curate target information for a therapeutic area, we examine the drugs indicated for the therapeutic area of interest and its descendants. The two therapeutic areas examined are rheumatoid arthritis (RA) and inflammatory bowel disease. For rheumatoid arthritis, we collected therapeutic data (i.e., targets of drugs indicated for the therapeutic area) from OpenTargets for rheumatoid arthritis (EFO_0000685), ankylosing spondylitis (EFO_0003898), and psoriatic arthritis (EFO_0003778). For inflammatory bowel disease, we collected therapeutic data for ulcerative colitis (EFO_0000729), collagenous colitis (EFO_1001293), colitis (EFO_0003872), proctitis (EFO_0005628), Crohn's colitis (EFO_0005622), lymphocytic colitis (EFO_1001294), Crohn's disease (EFO_0000384), microscopic colitis (EFO_1001295), inflammatory bowel disease (EFO_0003767), appendicitis (EFO_0007149), ulcerative proctosigmoiditis (EFO_1001223), and small bowel Crohn's disease (EFO_0005629).

We define positive examples (i.e., where the label y = 1$ as proteins targeted by drugs that have at least completed phase 2 of clinical trials for treating a certain therapeutic area. As such, a protein is a promising candidate if a compound that targets the protein is safe for humans and effective for treating the disease. We retain positive training examples that are activated in at least one cell type specific protein interaction network. The final number of positive training examples for RA and IBD are 152 and 114, respectively.

We define negative examples (i.e., where the label y = 0) as druggable proteins that do not have any known association with the therapeutic area of interest according to OpenTargets. A protein is deemed druggable if it is targeted by at least one existing drug. We extract drugs and their nominal targets from DrugBank. We retain negative training examples that are activated in at least one cell type specific protein interaction network. The final number of negative training examples for RA and IBD are 1,465 and 1,377, respectively.

Data exclusions	No data was excluded from the analysis.
Replication	Data on biological replicates were subject to statistical tests to ensure effects were significant. All replication attempts were successful. For the analyses of tissue hierarcy (Figure 3c), tissue ontology was shuffled 10 times to produce gray distribution. For analyses of RA/IBD models, ll the experiments were performed independently 10 times using different randomly-selected seeds.
Randomization	Randomization was performed using unbiased, non-parametric, random sampling and perturbation-based null hypothesis testing.
Blinding	Any group allocations were programmatically randomly generated and not assigned by the investigators, so blinding is not relevant to this study.

Reporting for specific materials, systems and methods

We require information from authors about some types of materials, experimental systems and methods used in many studies. Here, indicate whether each material, system or method listed is relevant to your study. If you are not sure if a list item applies to your research, read the appropriate section before selecting a response.

Materials & experimental systems

n/a	Involved in the study
☒	☐ Antibodies
☒	☐ Eukaryotic cell lines
☒	☐ Palaeontology and archaeology
☒	☐ Animals and other organisms
☒	☐ Clinical data
☒	☐ Dual use research of concern

Methods

n/a	Involved in the study
☒	☐ ChIP-seq
☒	☐ Flow cytometry
☒	☐ MRI-based neuroimaging
